# Dynamic Changes of Immunoreactive CD34, CD117, and CD41 Hematopoietic Stem Cells in Human Placentas of Different Gestational Ages

**DOI:** 10.3390/jdb13020016

**Published:** 2025-05-09

**Authors:** Sanja Jovicic, Ivan R. Nikolic, Ljiljana Amidžić, Vesna Ljubojevic, Maja Barudzija, Ranko Skrbic

**Affiliations:** 1 Centre for Biomedical Research, Faculty of Medicine, University of Banja Luka, 78000 Banja Luka, Bosnia and Herzegovina; ljiljana.amidzic@med.unibl.org (L.A.); maja.barudzija@med.unibl.org (M.B.); ranko.skrbic@med.unibl.org (R.S.); 2 Department of Histology and Embryology, Faculty of Medicine, University of Banja Luka, 78000 Banja Luka, Bosnia and Herzegovina; vesna.ljubojevic@med.unibl.org; 3 Department of Histology and Embryology, Faculty of Medicine, University of Niš, 18000 Niš, Serbia; inikolic@junis.ni.ac.rs; 4 Department of Human Genetics and Cell Biology, Faculty of Medicine, University of Banja Luka, 78000 Banja Luka, Bosnia and Herzegovina; 5 Ophtalmology Clinic, University Clinical Center Banja Luka, 78000 Banja Luka, Bosnia and Herzegovina; 6 Department of Pharmacology and Toxicology, Faculty of Medicine, University of Banja Luka, 78000 Banja Luka, Bosnia and Herzegovina

**Keywords:** hematopoiesis, hematopoietic stem cells, human placenta

## Abstract

**Background**: The process of prenatal hematopoiesis occurs in various anatomical locations, including the placenta. The placenta is not merely a temporary hematopoietic reservoir, but it is one of the key sites for the synthesis of hematopoietic stem cells (HSCs). This study aimed to investigate the presence, distribution, and immunoprofiles of HSCs in the human placenta during different gestational periods. **Materials and Methods:** Placental samples of different gestational ages (first, second, and third trimesters) were analyzed using classical hematoxylin and eosin staining and immunohistochemical staining for CD34, CD117, and CD41 markers, with HSC quantification through numerical areal density (N_A_). **Results:** Highly immunoreactive CD34 HSCs were present in placentas throughout gestation, while highly immunoreactive CD117 and CD41 HSCs were observed during the first two trimesters. In the first trimester, HSCs were found within the lumen of blood vessels and as individual cells in the mesenchyme of chorionic villi. With advancing gestation, the number of HSCs in the mesenchyme of chorionic villi increased. **Conclusions:** Immunoreactive CD34, CD117, and CD41 cells are present in significant proportions in various parts of the placenta throughout gestation, indicating that the placenta provides a substantial proportion of HSCs for hematopoiesis.

## 1. Introduction

Hematopoiesis is the process of blood cell formation. Hematopoietic stem cells (HSCs) sustain the blood system by generating blood cells of all lineages through multipotent progenitors [1,2]. During prenatal life, the hematopoietic system has the following two key roles: rapidly generating mature blood cells essential for fetal growth and development and establishing a reservoir of HSCs required for postnatal life. These cells have the ability to self-renew and differentiate into two types of multipotent cells, myeloid and lymphoid. Their division can produce either multipotent cells or differentiated unipotent stem cells, which further divide and differentiate into morphologically recognizable cells of a single lineage [3,4].

In postnatal life, hematopoiesis occurs exclusively in the bone marrow, but during prenatal development, it takes place at different anatomical sites across three waves [5,6].

The first wave begins extraembryonically, around day 7 of embryogenesis, in the yolk sac, generating primitive red blood cells, macrophages, and a few megakaryocytes. Blood islands form in the extraembryonic mesoderm, producing these primitive blood cells [7]. The second wave, definitive hematopoiesis independent of HSCs, also starts in the yolk sac around day 8 of embryonic development. Lymphomyeloid progenitors and erythromyeloid progenitors migrate to the fetal liver, supporting embryonic survival during mid-gestation [8]. The third wave, HSC-dependent hematopoiesis, occurs in the aorta–gonad–mesonephros region, initially in the dorsal aorta and later in major arteries (vitelline and umbilical arteries) and the chorionic plate mesenchyme of the placenta. From these locations, HSCs migrate to temporary hematopoietic niches, such as the liver, placenta, spleen, and bone marrow [9]. In 2005, researchers proposed that the placenta is not just a reservoir of HSCs derived from other locations but also a site of hematopoiesis [10,11].

The placenta, a temporary organ essential for normal fetal growth and development, is unique in consisting of tissues of dual origin—maternal and fetal. Its role is critical in fetal development, with numerous functions. Placental development begins around days 7–8 post-fertilization with the implantation of the blastocyst into the decidually transformed endometrium, influenced by pro-inflammatory cytokines and prostaglandin E3 [12].

It is hypothesized that the development of placental blood vessels parallels HSC development, as endothelial cells and HSCs share common progenitors. The hematopoietic microenvironment of the placenta is a dynamic and multifaceted niche that supports early blood cell development. It includes various cells, extracellular matrix components, growth factors, cytokines, and adhesion molecules, creating a specialized environment that enables HSC proliferation, differentiation, and maintenance during critical fetal development stages. This microenvironment promotes HSC proliferation while preventing premature differentiation into various cell lineages [13].

Hematopoietic stem cells exhibit morphological characteristics similar to small- or medium-sized lymphocytes, with diameters of 7–8 µm. They possess a centrally located round nucleus surrounded by a cytoplasmic rim. Identification relies on surface-expressed markers, such as CD34 and CD117, which have been widely used for detecting hematopoietic stem cells [4,13].

HSC types in the placenta present a challenge in distinguishing HSCs originating in the placenta from those arriving via circulation from other hematopoietic niches. Studies using Runx1-lacZ and Runx1+/− knockout mice, which lack cardiac activity and blood flow to the placenta, have identified CD41 immunoreactive HSC populations [14].

CD34 is a transmembrane phosphoglycoprotein initially identified on HSCs and progenitor cells, including pluripotent hematopoietic stem cells (PHSCs) and colony-forming units (CFU-GEMM). CD34 expression is almost universally associated with hematopoietic cells and is also found on endothelial cells and embryonic fibroblasts [15,16,17].

CD117 (c-kit) is a class III tyrosine kinase transmembrane receptor for stem cell factor, encoded by the proto-oncogene c-kit. It identifies human HSC populations with high proliferation and self-renewal potential and is expressed on germ cells, melanocytes, mast cells, and interstitial cells of Cajal [18,19].

CD41 (integrin αIIb) is a heterodimeric integral membrane protein and a marker for early embryonic HSCs, and it is also expressed on platelets and megakaryocytes [20].

Using placentas post-delivery in regenerative medicine is a current trend due to their availability and the abundance of stem cells and growth factors they contain. While the placenta’s role in hematopoiesis is established, the exact localization, distribution, and differentiation potential of HSCs isolated from the placenta remain incompletely defined.

This study aims to determine the presence of hematopoietic stem cells in placentas of different gestational ages, their localization within the placenta, and their abundance using numerical areal density (N_A_), to evaluate the placenta as a suitable source for HSCs isolation.

## 2. Materials and Methods

### 2.1. Placenta Sampling

This study was conducted in accordance with the latest revision of the Helsinki Declaration, with approval from the Ethics Committee (No: 18/4.167/21). Placental samples were collected at the Clinic for Gynecology and Obstetrics and the Pathology Department of the University Clinical Center of the Republika Srpska (UCC RS). Placentas were sampled in all periods of gestation in the first, second, and third trimesters (Table 1). In the human population, pregnancy lasts 40 weeks and is divided into trimesters, the first lasting from 0–13 weeks of gestation, the second up to 14–27 weeks of gestation, and the third trimester from 28 to 40 weeks of gestation. Placentas of the first trimester were sampled at the Department of Pathology of the UCC RS, as part of intratubal pregnancies that were referred to for pathohistological verification after fallopian tube rupture. Already existing pathohistological paraffin molds from the department’s collection were used, and all parts of the placenta were preserved where there was no coagulum and inflammatory infiltrate that could interfere with the visualization and analysis of the placental tissue. Placentas of the second trimester were sampled after premature births within this gestation. Only samples without macroscopically visible deviations were included in this study. Placentas of the third trimester were sampled at the Clinic for Gynecology and Obstetrics, after vaginal delivery. Our recent study demonstrated that advanced maternal age impacts placental morphology [21]. Therefore, all samples were obtained from healthy pregnant women under 35 years old, with no history of chronic non-communicable diseases, infections, or smoking.

### 2.2. Tissue Processing

Samples from ectopic pregnancies included the entire circumference of the fallopian tube. Second- and third-trimester samples were 2 × 2 cm in size, covering the full thickness of the placenta from the chorionic to basal plate. After 48 h of fixation in 4% formaldehyde, the samples were processed in a Leica TP1020 tissue processor, embedded in paraffin blocks, sectioned into 4 µm slices, and stained using hematoxylin-eosin and immunohistochemistry for CD34 (anti-CD34 monoclonal antibody, 1:100, Abcam, Cambridge, UK), CD117 (c-Kit monoclonal antibody, 1:100, Invitrogen, Waltham, MA, USA), and CD41 (rabbit polyclonal anti-CD41 antibody, 1:200, Invitrogen). Antigen retrieval was performed by heating in citrate buffer (pH 6) for 20 min. Endogenous peroxidase activity was blocked with 3% hydrogen peroxide. Nonspecific background staining was blocked using UltraVision Block (Thermo Fisher Scientific, Fremont, CA, USA). Primary antibodies were incubated at room temperature for 30 min, and visualization was performed using the HRP/DAB IHC detection system (Abcam). Counterstaining was performed with Mayer’s hematoxylin. Analyses were conducted using a Leica DM6000 microscope equipped with a Leica DFC310FX camera.

The intensity of immunoreactivity for CD34, CD117, and CD41 was semi-quantitatively graded as low (+), moderate (++), or high (+++).

### 2.3. Morphometric Analysis

In order to quantify CD34, CD117, and CD41 immunopositive hematopoetic cells, we determined their numerical areal density and the average number of cells in 1 mm^2^ of tissue (N_A_) with the ImageJ software (version 18.0). Numerical areal density represents the number of analyzed cells, CD34, CD117, and CD41 immunoreactive cells, relative to the surface area of the field of view. Numerical areal density was calculated using the formula N_A_ = N/A, where N is number of analyzed immunoreactive cells (N), and A is the field of view area.

The number of examined fields of view (N) was determined using the formula N = (20 × SD/X)^2^, where SD is the standard deviation and X is the mean value of results obtained in a pilot study conducted on 20 fields of view (Kališnik M, 2002) [22].

### 2.4. Statistical Analysis

The statistical analysis of the collected data was performed using the R 4.2.3 statistical software package.

Descriptive statistics were used to determine frequencies, measures of central tendency, and measures of variability and for the graphical representation of the results.

The Chi-square test (χ2, Chi-square Test) was used to compare the frequencies of occurrence of categorical variables in independent samples.

The statistical analysis of numerical data and the selection of an appropriate test depended on the distribution of numerical data. The normality of distribution was determined based on skewness values (from −3 to +3 indicating normal distribution) and kurtosis values (from −1 to +1 indicating normal distribution), as well as the Shapiro–Wilk test.

Student’s *t*-test (*t*-test for two independent samples) was used to compare the mean values of two independent samples with a normal distribution of numerical data. In cases of deviation from normal distribution, the Mann–Whitney U test was applied for two samples.

The homogeneity of variances for more than two groups was tested using Levene’s test. After statistical analysis and the application of Levene’s test, ANOVA (*p* > 0.05 from Levene’s test) or the Kruskal–Wallis Test (*p* < 0.05 from Levene’s test) was used.

All results were considered statistically significant if *p* ≤ 0.05 and highly statistically significant if *p* < 0.001. In cases where highly statistically significant results were obtained, the level of statistical significance (<0.001) was reported.

## 3. Results

The differences in placental structure across various gestational periods are most pronounced in the composition of the chorionic villi. In first-trimester placentas, up to the 10th week of gestation, mesenchymal chorionic villi are the most abundant, along with a few immature intermediate villi. The number of immature intermediate villi increases after the 10th week of gestation. During the second trimester, mature intermediate villi dominate, while in the third trimester, both mature intermediate and terminal villi are present.

**Figure 1:**
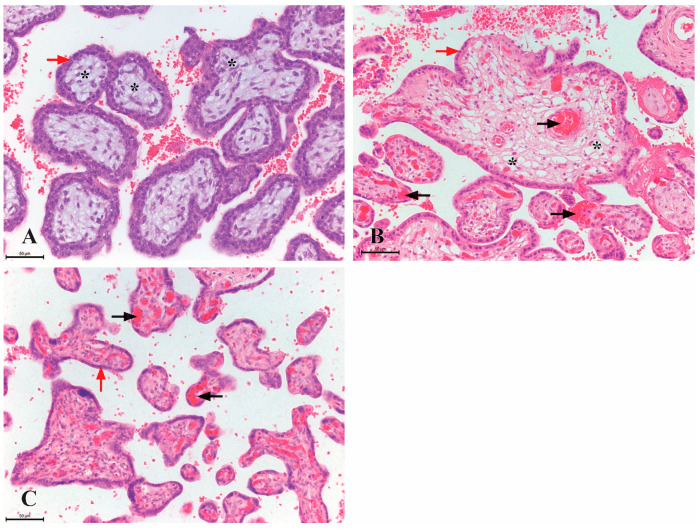
Histological structure of chorionic villi in the human placenta (H&E staining, ×200, scale bar 50 µm). (**A**) The mesenchymal type of chorionic villi from first-trimester placentas (8th week of gestation); the villi are predominantly composed of mesenchymal connective tissue (marked with a star) and are encased by two distinct layers of trophoblastic cells (red arrow), and no blood vessels are evident within the stroma. (**B**) The intermediate type of chorionic villi from second-trimester placentas (23rd week of gestation); the stroma contains numerous stromal channels (indicated by a star) and blood vessels filled with erythrocytes (black arrow), these structures are surrounded by a single layer of trophoblastic cells (red arrow). (**C**) Terminal chorionic villi from third-trimester placentas (36th week of gestation); the stroma is densely vascularized, containing numerous blood vessels (black arrow), while the surface is covered by a continuous layer of syncytiotrophoblasts (red arrow).

The mesenchymal type of villi consists of mesenchymal stroma with sparse blood vessels, whose lumen is not visible. The stroma is surrounded by two layers of trophoblastic cells, which are cytotrophoblasts and syncytiotrophoblasts. Immature intermediate villi contain numerous stromal channels within their stroma, formed by the merging of cytoplasmic extensions from stromal cells. Numerous blood vessels are observed within the stroma. On the surface of the villi, the syncytiotrophoblast layer is more pronounced, with individual cytotrophoblastic cells located beneath it. In intermediate and terminal villi, the number of blood vessels within the stroma increases, while only the syncytiotrophoblast is observed on the surface (Figure 1).

**Figure 2:**
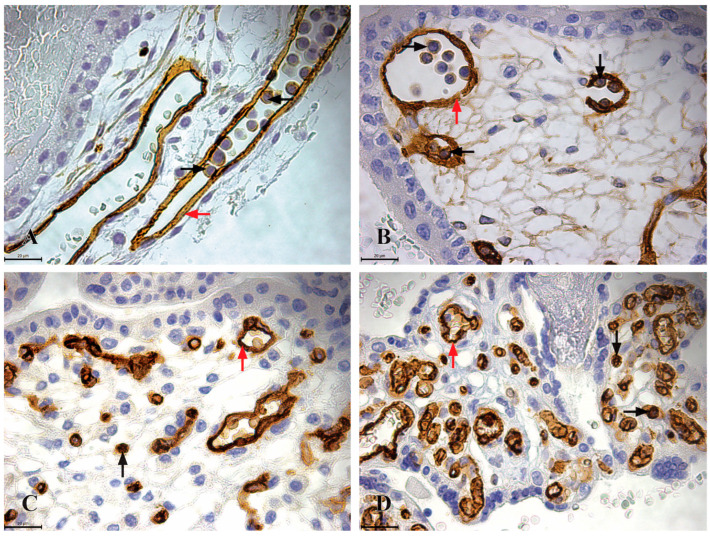
CD34 Immunoreactivity in the chorionic plate and chorionic villi of human placentas from the first, second, and third trimesters (×630, scale bar 20 µm). (**A**) Chorionic plate of a first-trimester placenta (11th week of gestation), where highly immunoreactive CD34 HSCs (+++) are clustered within the lumen of blood vessels (black arrow), and endothelial cells within the blood vessels of the chorionic plate exhibit strong CD34 immunoreactivity; (**B**) chorionic villi of a first-trimester placenta (11th week of gestation), with highly immunoreactive CD34 HSCs (+++) grouped in clusters within the lumen of blood vessels; (**C**) chorionic villi of a second-trimester placenta (22nd week of gestation), where highly immunoreactive CD34 HSCs (+++) are located as individual cells in the mesenchyme of the chorionic villi (black arrow), and endothelial cells also display strong CD34 immunoreactivity (red arrow); (**D**) chorionic villi of a third-trimester placenta (36th week of gestation), where highly immunoreactive CD34 HSCs (+++) are present as individual cells in the mesenchyme of the chorionic villi (black arrow), while endothelial cells continue to exhibit strong CD34 immunoreactivity (red arrow).

The CD34 immunoreactive cells are present in placentas across all trimesters, exhibiting consistent morphology throughout. These cells are approximately 7 µm in size, round in shape, with a centrally located round nucleus surrounded by a variable amount of cytoplasm. During the first trimester, highly immunoreactive CD34 hematopoietic stem cells (+++) are abundant, appearing as clusters within the lumen of blood vessels in the chorionic plate and chorionic villi. In the second-trimester placenta samples, the CD34 immunoreactive cells are observed within the mesenchyme of placental villi as individual cells. In the third-trimester placenta samples, the highly immunoreactive CD34 hematopoietic stem cells are located in the mesenchyme of villi and the chorionic plate. However, these cells are absent from the lumen of blood vessels (Figure 2).

The mean values of N_A_ of CD34 immunoreactive HSCs at different gestational ages are shown in Table 2. A significant difference was observed between the means of the second and third trimesters (*p* = 0.01). However, no statistically significant difference was found between means of the first and second trimesters (*p* = 0.6) or between means of the first and third trimesters (*p* = 0.07).

Highly immunoreactive CD34 expression (+++) was also observed in endothelial cells of placental blood vessels (Figure 2).

**Figure 3:**
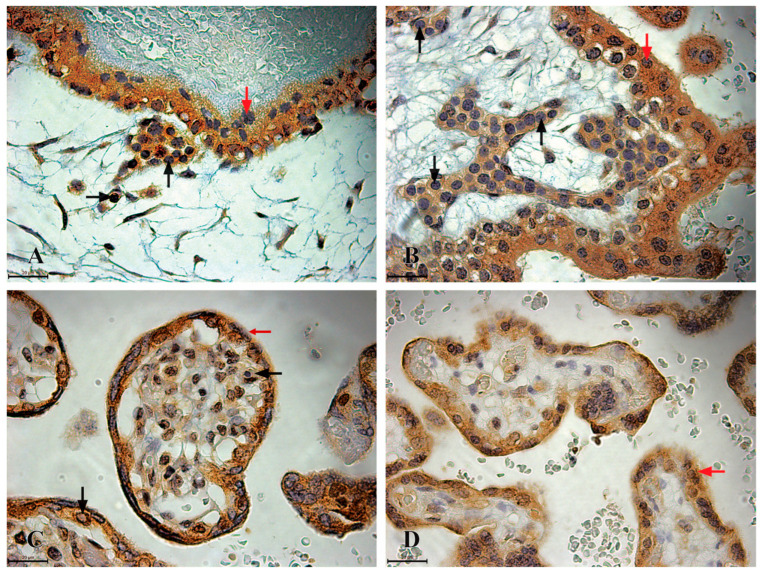
CD117 immunoreactivity in the chorionic plate and chorionic villi of human placentas from the first, second, and third trimesters. (**A**) The chorionic plate of a first-trimester placenta (11th week of gestation), where highly immunoreactive CD117 HSCs (+++) are clustered within the lumen of blood vessels (black arrow), and trophoblastic cells exhibit strong CD117 immunoreactivity (red arrow); (**B**) the chorionic villi of a first-trimester placenta (11th week of gestation), where highly immunoreactive CD117 HSCs (+++) completely fill the lumen of a blood vessel; (**C**) the chorionic villi of a second-trimester placenta (22nd week of gestation), where highly immunoreactive CD117 HSCs (+++) are present as individual cells within the mesenchyme of the chorionic villi (black arrow), and trophoblastic cells also display strong CD117 immunoreactivity (red arrow); (**D**) the chorionic villi of a third-trimester placenta (36th week of gestation), where only trophoblastic cells demonstrate moderate CD117 immunoreactivity (++), which is indicated by the red arrow, while immunoreactive CD117 HSCs are absent.

Highly immunoreactive CD117 cells (+++), morphologically resembling HSCs, are present in the placentas of the first and second trimesters. These cells, similar to CD34-positive cells, are approximately 7 µm in size, round in shape, with a centrally located round nucleus surrounded by a variable amount of cytoplasm (Figure 3). In the first trimester placenta sample the highly immunoreactive CD117 HSCs cells can be found in clusters within the lumen of blood vessels in the chorionic plate and villi, as well as individually within the mesenchyme of villi. In the second trimester, these cells can be observed only within the mesenchyme of chorionic villi (Figure 3).

Trophoblastic cells, including cytotrophoblasts and syncytiotrophoblasts, express CD117 immunoreactivity in all trimesters, although its intensity decreases with advancing gestation. Cytotrophoblasts are uniform, cuboidal cells with a central nucleus and abundant cytoplasm. They exhibit high CD117 immunoreactivity (+++) in the first trimester but low reactivity (+) in the second and third trimesters. Syncytiotrophoblasts are irregularly shaped, variably sized cells. These show high CD117 immunoreactivity (+++) in the first and second trimesters, which decreases to moderate reactivity (++) in the third trimester (Figure 3).

The medians of N_A_ of CD117 immunoreactive HSCs at different gestational ages are shown in Table 3. No significant statistical difference was found in the medians between the first and second trimesters (*p* > 0.05).

**Figure 4:**
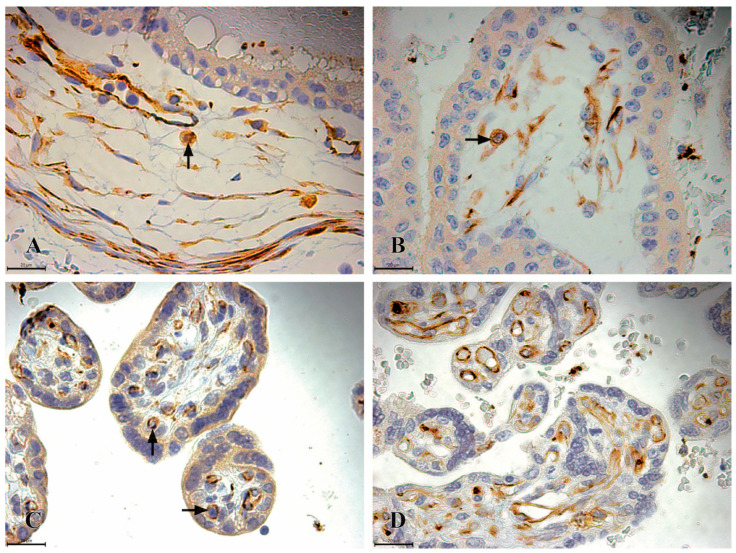
CD41 immunoreactivity in the chorionic plate and chorionic villi of human placentas from the first, second, and third trimesters. (**A**) The chorionic plate of a first-trimester placenta (11th week of gestation), where highly immunoreactive CD41 HSCs (+++) are observed as individual cells located in the mesenchymal connective tissue of the chorionic plate (black arrow); (**B**) the chorionic villi of a first-trimester placenta (11th week of gestation), with highly immunoreactive CD41 HSCs (+++) observed as individual cells situated in the mesenchymal connective tissue; (**C**) the chorionic villi of a second-trimester placenta (22nd week of gestation), where highly immunoreactive CD41 HSCs (+++) are located as individual cells in the mesenchyme of the chorionic villi (black arrow); (**D**) in the chorionic villi of a third-trimester placenta (36th week of gestation) CD41 immunoreactive cells are not present.

CD41 immunoreactive cells first appear after the 11th week of gestation up to 22 weeks of gestation, and they are localized exclusively within the mesenchyme of chorionic villi. These cells exhibit high immunoreactivity (+++) and share the same morphological features as CD34 and CD117-positive cells. In the second trimester, the number of CD41 cells decreases while maintaining their first-trimester localization. In the third-trimester placenta samples, the CD41 immunoreactive cells are absent (Figure 4).

The mean value of N_A_ of CD41 immunoreactive HSCs at different gestational ages are shown in Table 4. No significant statistical difference was found in the means between the first and second trimesters (*p* > 0.05) (Table 4).

In order to determine whether there are differences in the concentrations of various immunophenotypes of HSCs cells in the first and second trimesters, we compared the mean values of N_A_ CD34, CD117, and CD41 immunoreactive cells in the first and second trimesters. The mean values of N_A_ of CD34 immunoreactive cells were statistically significantly higher compared to the mean values of Na CD117 and CD41 immunoreactive cells in the first trimester, (*p* < 0.05). In the second trimester, the mean values of N_A_ of CD34 immunoreactive cells were also higher than the mean values of N_A_ of CD117 and CD41 immunoreactive cells. The difference between the groups in the second trimester was statistically significant (*p* ≤ 0.001), (Table 5).

## 4. Discussion

The placenta represents a rich source of hematopoietic stem cells with diverse profiles and phenotypes. Besides functioning as a temporary hematopoietic niche, it is also a primary site of HSC development during embryogenesis [11,23]. HSCs are present in the placenta throughout the entire gestational period [24]. Our study confirmed the presence of CD34 immunoreactive HSC populations throughout gestation, while CD117 immunoreactive cell populations were found between the 7th and 24th weeks of gestation, and CD41 immunoreactive cell populations were found only between the 11th and 22nd weeks of gestation.

The CD34 immunoreactive cell population was the most abundant, reaching its peak during the second trimester. Similarly, the populations of CD117 and CD41 cells were most numerous in the second trimester, suggesting the migration of HSCs from the placenta to other hematopoietic niches by the end of this period.

After the 24th week of gestation, the presence of CD34 immunoreactive HSCs in the placenta decreases significantly, coinciding with the establishment of hematopoiesis in the fetal liver and bone marrow, which become the main hematopoietic sites during later gestation [25]. During the first two trimesters, the placenta exhibits higher N_A_ of CD34 and CD117 cells compared to the fetal liver, whereas these values significantly increase in the liver after the 24th week of gestation. The advantages of using HSCs from the placenta for therapeutic purposes, as compared to traditional sources, are primarily rooted in their availability. Therefore, the discovery of a population of CD34 immunoreactive cells in term placentas is highly significant.

Although CD34 is a key marker for blood progenitor cells, it is also expressed by endothelial cells and embryonic fibroblasts. However, based on the morphology of the cells, their location, and the intensity of immunopositivity, it can be confidently concluded that these cells are the HSCs. The quantification of immunoreactive cells using flow cytometry often provides total concentrations of all CD34-expressing cells, including endothelial cells and fibroblasts, which may explain the discrepancy between our results and those of other studies [15,26,27,28]. Earlier studies have emphasized that HSCs are restricted to the chorion and chorionic villi [29]. However, we observed that, in addition to being located in the mesenchyme of the chorionic villi, populations of CD34 and CD117 immunoreactive cells were also present within blood vessels. We hypothesize that HSCs within the blood vessels are not primarily generated in the placenta but are transported there via blood from other hematopoietic niches. In contrast, cells localized in the mesenchyme of the placental villi likely originate in the placenta from hemangioblasts or mesenchymal progenitors. Supporting this is the finding that CD41 immunoreactive cells are exclusively present in the mesenchyme of chorionic villi, which was previously confirmed to be a marker of HSCs generated in the placenta [14].

This dual origin of HSCs in the placenta may also explain the differences in N_A_ values for CD34 and CD117 populations. It is believed that CD117 immunoreactive cells originate from mesenchymal progenitors, while CD34 immunoreactive cells are derived from hemangioblasts [30,31]. Since the N_A_ values of CD34 immunoreactive cells are higher compared to the other populations studied, it can be concluded that placental HSCs are predominantly of hemangioblast origin. The labyrinthine vasculature, along with mesenchymal stem cells (MSCs), which are also part of the placental niche, provides the necessary conditions for HSC self-renewal, proliferation, and differentiation. This is reflected in the higher abundance of HSCs in the mesenchyme compared to the lumen of blood vessels in the placenta [13,32].

The majority of HSCs in blood vessels differentiates into erythrocytes by the 14th week of gestation to ensure adequate oxygen supply to the growing fetus. The placenta is likely the primary site where erythrocyte maturation begins, including the loss of erythroblast nuclei under the influence of mesenchymal and Hofbauer cells [33].

The results of our study indicate that, in addition to hematopoietic progenitors, trophoblast cells also exhibit CD117 immunoreactivity, with trophoblasts of the first-trimester placenta showing high levels of immunoreactivity. However, the intensity of immunoreactivity decreases as gestation progresses. Since CD117 is a marker for stem progenitor cells, these trophoblast cells possess a high capacity for proliferation and differentiation. Their numbers, as well as their ability to proliferate and differentiate, decrease as pregnancy advances [34].

It is important to note that the identified HSCs in the placenta have the potential for proliferation, indicating that they are mature cells and can be used as transplants. Populations of CD34 and CD117 immunoreactive cells have the ability for multilineage and definitive differentiation [4,29,35,36].

The placenta has long been recognized as a significant biological material in regenerative medicine. However, the application of placental stem cells remains largely at the level of clinical studies. Due to its availability and size, the term placenta could provide a greater quantity of hematopoietic stem cells compared to traditional sources such as bone marrow-derived HSCs. Additionally, one of the first published reports on successful HSC transplantation from the placenta highlighted its potential application in unrelated recipients without triggering adverse reactions, such as graft-versus-host disease (GVHD). GVHD, which remains the most significant complication and leading cause of mortality following allogeneic hematopoietic stem cell transplantation [37]. Protocols have been developed that incorporate the use of placental MSC in GVHD therapy [38,39]. Placental MSCs are also utilized to stimulate the proliferation and differentiation of HSCs derived from other sources [40]. This direct interaction, specifically the stimulation of HSCs by placental MSCs, could be one of the key reasons to prioritize placenta-derived HSCs. Beyond their direct transplantation potential, placental HSCs can differentiate into various cell lineages. Notably, natural killer (NK) cells derived from placental HSCs have shown promising results not only in lymphoma treatment but also in combating viral infections resistant to conventional therapies [41,42]. However, despite these advantages, the isolation of HSCs from the placenta remains more complex compared to traditional sources, presenting a challenge for widespread clinical application.

## 5. Conclusions

The placenta is a dynamic hematopoietic microenvironment that contains diverse populations of hematopoietic stem cells. Immunoreactive cells expressing CD34, CD117, and CD41 are present in various regions of the placenta throughout gestation, contributing significantly to hematopoiesis.

Since the term placenta is available after birth, it can serves as a valuable source of HSCs. Future research should focus on optimizing conditions for the clinical application of HSCs isolated from the placenta.

## Figures and Tables

**Table 1:** The number of samples included in the study, allocated to different groups based on trimesters of development and weeks of gestation.

Development Period	WGA	Placentas N
First Trimester N = 14	7	2
	8	2
	9	3
	10	1
	11	4
	12	2
Second Trimester N = 12	19	5
	20	5
	23	2
Third Trimester N = 10	28	1
	35	1
	36	5
	37	3

WGA—weeks of gestational age, N—number of placentas of a given gestational age.

**Table 2:** The mean value and standard deviations of N_A_ of CD34 immunoreactive HSCs in human placenta in three trimesters.

Development Period	Mean	SD	*p*
First Trimester	409.9	244.3	0.04 *
Second Trimester	462.5	174.8	
Third Trimester	249.3	59.8	

Data are expressed as mean ± SD; * statistical significance, *p* ≤ 0.05, ANOVA.

**Table 3:** The values of N_A_ of CD117 immunoreactive HSCs in human placentas in three trimesters.

Development Period	Median	IQR	*p*
First Trimester	222.2	118.2	0.18
Second Trimester	187.5	23.8	
Third trimester	0	0	

Data are expressed as median (interquartile range: IQR), Man–Vitni test.

**Table 4:** The values of N_A_ of CD41 immunoreactive HSCs in human placentas in three trimesters.

Development Period	Mean	SD	*p*
First Trimester	50.9	11.16	0.46
Second Trimester	56.2	12.7	
Third Trimester	0	0	

Data are expressed as mean ± standard deviations (SD), Student *t* test.

**Table 5:** Differences in N_A_ values among various types of HSCs during the first and second trimesters.

	First Trimester			Second Trimester		
HSCs	Mean	SD	*p*	Median	IQR	*p*
CD34	409.9	244.3	0.003 *	462.5	174.8	0.0002 **
CD117	267.5	145.8		187.5	23.8	
CD41	50.9	11.6		54.9	18.4	

Data are expressed as median (interquartile range, IQR), mean, and standard deviation (SD); * statistical significance, *p* ≤ 0.05; ** very high statistical significance, *p* < 0.001, Kruskal–Wallis test, ANOVA.

## Data Availability

All data supporting the findings can be found within the manuscript.
